# Cytomegalovirus and Hemolytic Anemia in an Immunocompetent Adult

**DOI:** 10.7759/cureus.31744

**Published:** 2022-11-21

**Authors:** Diana Ferrão, Clara Silva, Luis Nogueira-Silva, Jorge Almeida

**Affiliations:** 1 Internal Medicine, Centro Hospitalar Universitário de São João, Porto, PRT; 2 Center for Research in Health Technologies and Information Systems (CINTESIS), Faculty of Medicine, University of Porto, Porto, PRT; 3 Department of Medicine, Faculty of Medicine, University of Porto, Porto, PRT

**Keywords:** autoimmunity, immunocompetent host, choluria, cytomegalovirus, hemolytic anemia

## Abstract

Hemolytic anemia is an increasingly recognized complication of cytomegalovirus (CMV) infection in immunocompetent patients. Although it is thought to be immune-mediated, other mechanisms have been proposed. The decision to treat is controversial but it may include antiviral and immunosuppressive therapy. We report a case of CMV-induced hemolytic anemia in a previously healthy 55-year-old woman. The patient presented with asthenia and choluria, and laboratory tests showed severe anemia with hyperbilirubinemia and elevated lactate dehydrogenase. A diagnosis of hemolytic anemia was made. Structural, enzymatic, toxic, pharmacological, and neoplastic causes were excluded. The CMV immunoglobulin M was positive, with a negative direct antiglobulin test. The patient had an improvement in clinical and laboratory status without any treatment, and two months later she had a full recovery of the anemia. This case illustrates that CMV infection might be associated with severe organ damage in immunocompetent patients but has an overall good prognosis without any directed treatment.

## Introduction

Cytomegalovirus (CMV) is responsible for significant morbidity and mortality in patients with immune deficiencies such as acquired immunodeficiency syndrome, organ transplants, and the use of immunosuppressive therapy. In an immunocompetent host, CMV infection is generally asymptomatic or produces a mononucleosis-like syndrome that resolves spontaneously in a few days. However, in less common cases, organ-specific complications have been associated with CMV infection in previously healthy adults [1]. Multiple case reports have described hemolytic anemia (HA) as one of the potentially life-threatening consequences of the infection. Although the pathophysiologic mechanisms remain obscure, they may relate to immunologic activation and antibody cross-reactivity [2]. Here, we present a case of severe HA in a previously healthy patient with a history of recent CMV infection.

This article was previously presented as a meeting abstract at the 25th National Congress of Internal Medicine, in Portugal, on May 23, 2019.

## Case presentation

A 55-year-old woman with hypertension and no other relevant medical history was admitted to our hospital complaining of severe asthenia and choluria over the previous two weeks. She denied having abdominal or chest pain, nausea, vomiting, dyspnea, or urinary symptoms. On physical examination, the patient was pale, anicteric, and hemodynamically stable. No organomegaly or lymphadenopathy was noted. Laboratory test results are shown in Table 1.

**Table 1:** Laboratory tests.

Test	Result	Reference range
Hemoglobin (g/dL)	5.8	12.0–16.0
Mean corpuscular volume (fL)	104	87–103
Mean corpuscular hemoglobin (pg)	31.4	27–35
Red cell distribution width (%)	22.6	11–16
Corrected reticulocyte index (%)	8	0.5–2.5
Schizocytes (number)	Absent	Absent
Platelets (×10^9^/L)	282	150–400
Leukocytes (×10^9^/L)	6.66	4.0–11.0
Immature cells (no)	Absent	Absent
Creatinine (mg/dL)	0.57	0.51–0.95
Urea (mg/dL)	30	10–50
Lactate dehydrogenase (U/L)	284	135–225
Haptoglobin (mg/dL)	52	41–165
Total bilirubin (mg/dL)	2.68	<1.20
Iron (ug/dL)	106	49–151
Ferritin (ng/mL)	868.4	10.0–250.0
Folic acid (ng/mL)	5.0	2.2–17.5
Vitamin B12 (pg/mL)	352	187–883

The direct antiglobulin test (DAT) was negative. The abdominal ultrasound demonstrated normal sized spleen and liver. The patient received a two-unit red cell transfusion and was admitted for further studies.

All findings pointed to HA. The small degree of elevation of bilirubins associated with normal levels of haptoglobin suggested that hemolysis was occurring mainly in the extravascular space. The main causes of HA were searched. The patient lived in an urban environment without pets. She had lived in South Africa for a short period of time and returned to Portugal several years prior. She mentioned an upper respiratory tract infection the week before, with odynophagia and low-grade fever, which lasted for about seven days and resolved spontaneously. She did not practice any high-impact sports and did not consume fava beans or take any new medication, teas, or food supplements, or was exposed to heavy metals or excessive alcohol consumption. Structural causes of hemolysis were also excluded.

**Figure 1:**
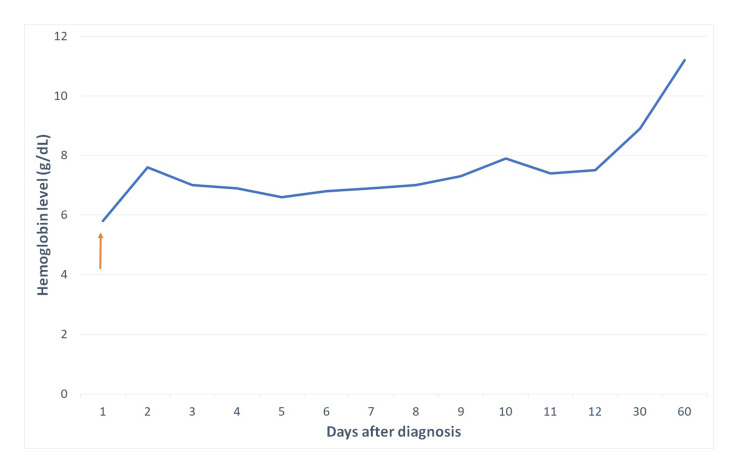
Hemoglobin level after the diagnosis. Hemoglobin level was stable after hospital admission but progressively increased one week after the diagnosis, reaching a level of 11.2 g/dL. This signals the spontaneous resolution of the infection and the cessation of the hemolytic process. The orange arrow represents a two-unit red blood cell transfusion at the emergency department.

The study of hemoglobin variants was normal, the peripheral blood smear had no aberrant erythrocytes. Enzymopathies were also excluded with normal values of CD55 and CD59, a negative essay for glucose-6-phosphate dehydrogenase deficiency and the absence of Heinz bodies. The hemolytic uremic syndrome was unlikely, with normal renal function and platelet count and no signs of microangiopathy. Serum protein electrophoresis revealed a slightly increased gamma globulin with a pattern suggestive of polyclonal gammopathy. Immunophenotyping demonstrated an increased immunoglobulin (Ig) G3 (IgG3); the other immunoglobulins were within range. Antinuclear antibodies were not significantly increased, with a titer of 1:100 with a speckled pattern, and the remainder of the autoimmune study was negative. There were no signs of complement consumption. The DAT test was repeated and was once again negative. Cryoglobulins were negative and cold agglutinins had a titer of 1:8 (for an accepted threshold of 1:64) [3,4]. A blood marrow biopsy was performed and showed erythrocyte hyperplasia with no morphological changes and without the involvement of other lineages. It further showed an inversion of the ratio of CD4/CD8 cells, highly suggestive of severe viral infection. The main viral infections were investigated. Serologies for human immunodeficiency virus, hepatitis, parvovirus B19, and Epstein-Barr virus were all negative. Anti-CMV IgM and IgG were positive, with an increased ratio of IgM to IgG, suggestive of recent infection; the DNA of CMV was negative. All things considered, a diagnosis of HA presumably caused by CMV infection was made. Throughout the following days, the patient had a spontaneous, yet slow, rise in serum hemoglobin (Figure 1), with progressively lower bilirubin and lactate dehydrogenase.

Because the infection was already in the seroconversion phase and hemolysis appeared to be improving, it was decided not to initiate antiviral or immunosuppressant therapy. Two months after discharge, the patient was asymptomatic and with an almost full recovery of the anemia, with a hemoglobin of 11.2 g/dL.

## Discussion

In the last few decades, several cases of severe CMV infection in immunocompetent adults have been reported. In a retrospective cohort of 124 patients with diagnosed CMV infection, Wreghitt et al. [5] concluded that organ damage not attributable to other causes was present in approximately 30% of patients. In a large systematic review, Rafailidis et al. [6] analyzed 89 studies with a total of 290 patients with immunological competence and severe CMV infection (defined as life-threatening or hospitalization requiring infection) and concluded that the main consequences of the infection were neurological, gastrointestinal, and hematological, with some cases of HA. Despite this, literature relating HA to CMV is sparse. Taglietti et al. [7] performed a review of cases describing HA caused by CMV in immunocompetent adults between 1998 and 2008 and found six reports, with a total of 12 patients. Of those, four had a positive DAT and three had a negative DAT; in the remaining five, the authors did not report the result of the DAT. Two were treated with corticosteroids alone, two received double treatment with corticosteroids and antiviral therapy, and three received no treatment. For the remaining five patients, we had no information about the treatment plan. All of the patients, despite the DAT result and the therapeutic strategy, had progressive improvement of the HA. In 2015, two more cases were published in the literature. The first [8] reported severe HA with thrombocytopenia, without signs of microangiopathy. DAT was positive and cold antibodies were identified with a titer of 1:256. Serology for CMV was positive. The second [9] reported bloody diarrhea associated with late-onset anemia with high lactate dehydrogenase levels. Serologies of CMV were positive and the patient was initiated on antivirals. Due to the persistence of anemia, he was later started on steroids, with an improvement in the clinical condition. The results of the DAT were not reported. A few months before the submission of this case, a similar case report was published [10] regarding a 22-year-old male with HA induced by CMV infection. There are a few substantial differences between that case and the one reported here. First, in the latter, the anemia was Coombs-positive, which highlights the fact that several pathophysiological mechanisms can underlie hemolysis in these patients. Second, the patient was started on intravenous Ig therapy, antiviral medication, and corticosteroids only after the stabilization of his hemoglobin level. The need for immunosuppressive therapy can relate to the presence of an autoimmune mechanism, demonstrated by the positive DAT, as opposed to the case presented here.

Here, we discuss the case of a 22-year-old male without significant medical history who presented with severe hemolytic anemia that required four units of packed red blood cells. Urinalysis showed microscopic hematuria but urine culture and drug screen reported normal findings. The hemoccult result at the bedside was negative. Abdominal ultrasound and computed tomography imaging all resulted in normal findings except for splenomegaly measured 18 cm. Hematology was consulted which showed a positive direct Coombs antibody test with 3+ IgG and 3+ complement. Peripheral blood smear showed no evidence of schistocytes or occasional teardrop cells but showed toxic granulations and neutrophils indicating an underlying infection. The patient underwent a bone marrow biopsy which showed erythroid hyperplasia with a slight increase in sideroblast cells but revealed no evidence of lymphoma, leukemia, or dysplasia. Infectious workup reported negative findings for human immunodeficiency virus and hepatitis panel. However, Epstein-Barr virus IgM antibodies to viral capsid antigen (VCA) had a value of greater than 160 U/mL. Polymerase chain reaction testing for CMV DNA detected high titers with 481,269 IU/mL. The patient initially received intravenous immunoglobulin therapy for five days, antiviral medication for seven days, and high-dose therapeutic corticosteroids resulting in the stabilization of his blood hemoglobin level.

The pathophysiological mechanisms underlying hemolysis in immunocompetent patients with CMV infection are not clear. It has been hypothesized that hemolysis is the result of an inappropriate immunologic activation and cross-reactivity antibodies that destroy red blood cells [7,11,12]. In some of the cases reported, including ours, the tests for autoimmunity, cold antibodies, and agglutinins were negative. It has, nonetheless, been recognized that some forms of autoimmune hemolytic anemia present with a negative DAT test. The exact incidence of DAT-negative autoimmune hemolytic anemia is not known, but it is thought to be around 3-11% [13-15]. Some possible explanations for the phenomenon include pre-analytic errors, lack of sensitivity of the DAT reagent used, loss of low-affinity antibodies on red cells during the pre-test washes, presence of IgM without complement fixation (because the DAT usually performed detects only IgG and C3), and cell-mediated autoimmunity with hyperactivity of natural killer cells [13,14,16]. Further tests can be performed to clarify the DAT negativity, including flow cytometry, enzyme-linked essays, and high-sensitivity antiglobulin tests [13]. These tests are not widely available and are not available at our hospital. In this particular case, also, the DAT test was performed at a later time due to bureaucratic difficulties, which can weaken the immune reaction [13]. Other pathogenic mechanisms have been proposed to be behind the hemolysis in CMV infection. A direct cytotoxic effect of CMV on both megakaryocytes and mature red blood cells has been found [17].

There is controversy regarding the pertinence and type of treatment. The decision to not initiate antiviral or immunosuppressant treatment in our case was based on the spontaneous improvement of our patient. In some of the cases described, treatment was initiated when the patient failed to improve [9]. In others, a wait-and-see approach was preferred [11]. A thorough review of the few studies published appears to suggest that clinical improvement occurs despite the decision to treat and the chosen treatment strategy [7].

This case has its limitations. First, the diagnosis was hindered due to the low sensitivity of the DAT test used in our hospital, which can yield false-negative results, and the late time at which it was performed. Second, the diagnosis made was one of exclusion. We concluded that the anemia was probably caused by CMV infection because all other hypotheses were ruled out. Because the patient improved on her own, we may never be absolutely certain of the CMV contribution to the hemolytic process.

## Conclusions

This and other case reports suggest that CMV infection may not be as innocent as previously thought and may be associated with severe cases of multisystemic disease, including HA. The organ-specific damage mediated by the virus appears to be self-limited, requiring only support measures in some cases, while in others directed treatment might be necessary. Nevertheless, the etiological diagnosis is essential, especially to rule out other relevant disorders that could underlie hemolysis. Moreover, the fact that CMV studies are not done regularly might underestimate this diagnosis. Further investigations are necessary to improve diagnostic and treatment methods for patients with atypical presentations of CMV infection.
